# Supplementary material for: Phenological Adaptation of Wheat Varieties to Rising Temperatures: Implications for Yield Components and Grain Quality
Source: Plants (Basel). 2024 Oct 18;13(20):2929. doi: 10.3390/plants13202929 (PMC11510792; doi:10.3390/plants13202929)
Supplement: Supplementary file 1 [file plants-13-02929-s001.zip › plants-3138172-supplementary.pdf]

Phenological adaptation of wheat varieties to rising temperatures: implications for yield components and grain quality

Davide Gulino[1], Marta S. Lopes[1*]

[1] Sustainable Field Crops Program, IRTA (Institute of Agrifood Research and Technology), Lleida, Spain

Corresponding author: Marta S. Lopes (marta.dasilva@irta.cat)

Supplementary material

Table S1. The results of a four-way factorial ANOVA for trials conducted in 2021 and 2022 are presented, with each crop variable analyzed. The relative sums of squares (reported as a percentage of the total sum of squares per factor) and the R^2 value for each model are also shown. Significant values are indicated in bold font ($p<0.05$) and in normal text when not significant ($p>0.05$).

**Factor**	**GDD (DH)**	**GDD (DM)**	**GF**	**GY**	**HLW**	**TKW**	**NG**	**Prot (%)**	**SDS**
R^2	0.99	0.96	0.97	0.94	0.96	0.91	0.93	0.95	0.93
Year	**0.5%**	**18.9%**	**56.7%**	**51.2%**	**17.9%**	**6.2%**	**57.5%**	**1.1%**	**47.7%**
Sowing	**1.0%**	**5.8%**	**8.8%**	**10.5%**	**3.1%**	**4.7%**	**7.7%**	**7.4%**	**7.5%**
Water	**1.4%**	**23.4%**	**9.9%**	**18.3%**	0.0%	**17.8%**	**14.7%**	**28.3%**	**12.7%**
Variety	**94.1%**	**47.8%**	**21.0%**	**9.4%**	**60.0%**	**32.5%**	**9.7%**	**23.1%**	**15.5%**
Year*Sowing	0.0%	**0.3%**	**0.7%**	**4.5%**	**2.0%**	**2.0%**	**2.8%**	0.0%	**0.2%**
Year*Water	**0.1%**	0.1%	0.0%	**0.7%**	**8.4%**	**17.1%**	0.1%	**29.8%**	**4.5%**
Year*Variety	**0.6%**	**0.6%**	**1.1%**	**2.5%**	**2.5%**	7.4%	**2.1%**	**2.0%**	**5.1%**
Sowing*Water	**0.3%**	**0.5%**	0.0%	0.0%	**1.6%**	**0.3%**	**0.2%**	**1.2%**	**1.0%**
Sowing*Variety	**1.2%**	0.3%	0.3%	**0.7%**	**0.7%**	**1.2%**	**1.2%**	0.4%	0.6%
Water*Variety	**0.1%**	**0.7%**	**0.4%**	0.5%	**1.3%**	**3.4%**	**0.9%**	**1.0%**	**2.2%**

Year*Sowing*Water	**0.1%**	**0.1%**	0.0%	0.0%	**0.5%**	**2.8%**	**0.2%**	**0.4%**	0.0%
Year*Sowing*Variety	**0.2%**	**0.4%**	0.2%	0.5%	0.3%	0.7%	0.5%	0.4%	0.6%
Year*Water*Variety	**0.2%**	0.2%	0.3%	**1.0%**	**0.6%**	**1.7%**	**1.7%**	**3.8%**	**1.9%**
Sowing*Water*Variety	**0.0%**	**0.5%**	**0.3%**	0.2%	**0.5%**	**1.1%**	0.7%	0.6%	0.3%
Year*Sowing*Water*Variety	**0.1%**	**0.4%**	0.3%	0.1%	**0.9%**	**1.1%**	0.3%	0.4%	0.3%

GDD (DH): growing degree days to heading (°Cd), GDD (DM): growing degree days to maturity (°Cd), GF: days of grain filling (days), GY: grain yield (t ha^{-1}), HLW: hectoliter weight (kg/hl), TKW: thousand kernel weight (g/1000 kernels), NG: number of grains m^{-2} (units m^{-2}), Prot (%): grain protein concentration (%), SDS: sodium dodecyl sulphate sedimentation (ml).

Table S2. ANOVA tests for trials conducted in Lleida 2021 and 2022 with ten wheat varieties, two water regimes (100% irrigated and rainfed), and two sowing dates per each crop variable with its relative sums of squares (reported as a % of total sum of square per each factor) and R^2 of each model are shown. Significant values are indicated in bold font ($p<0.05$) and in normal text when not significant ($p>0.05$).

**Factor**	**W**	**P/L**	**P**	**L**
R^2	0.65	0.09	0.57	0.31
Year	**85.7%**	42.0%	**89.9%**	**50.6%**
Sowing	0.9%	0.3%	0.1%	3.0%
Water	**8.5%**	19.1%	**9.3%**	6.2%
Year*Sowing	0.1%	0.0%	0.0%	0.4%
Year*Water	**3.9%**	37.6%	0.0%	**34.5%**
Sowing*Water	0.6%	0.1%	0.3%	1.4%
Year*Sowing*Water	0.4%	0.9%	0.5%	4.0%

W: dough strength (10^{-4} J), P/L: tenacity/extensibility ratio, P: tenacity (mm), L: extensibility (mm).

Table S3. Four-way factorial ANOVA for trials conducted in Lleida 2021 and 2022 per each weather variable measured during the vegetative (V) and grain filling stages (GF) with relative sums of squares (reported as a % of total sum of square per each factor) and R^2 of each model are shown. Significant values are indicated in bold font ($p<0.05$) and in plain text when not significant ($p>0.05$).

Factor	Tmin V	TA V	TM V	PP V*	SR V	ETo V	Tmin GF	TA GF	TM GF	PP GF*	SR GF	ETo GF
R^2	0.99	0.99	0.99	0.99	0.99	0.99	0.98	0.99	0.99	0.99	0.97	0.95
Year	**6.4%**	**0.3%**	**14.5%**	**52.9%**	**6.2%**	**2.5%**	**24.0%**	**52.2%**	**73.9%**	**84.4%**	**54.1%**	**40.9%**
Sowing	**25.8%**	**46.3%**	**49.7%**	**13.8%**	**0.7%**	**3.9%**	**8.3%**	**6.6%**	**4.1%**	**3.4%**	**6.1%**	**5.7%**
Water	**1.0%**	**0.7%**	**0.4%**	**0.2%**	**1.4%**	**1.4%**	**7.1%**	**4.4%**	**2.3%**	**0.0%**	**13.4%**	**26.5%**
Variety	**63.5%**	**49.1%**	**30.8%**	**15.4%**	**88.7%**	**89.2%**	**58.1%**	**35.3%**	**18.6%**	**5.3%**	**21.7%**	**19.9%**
Year*Sowing	**0.3%**	**0.3%**	**1.9%**	**4.9%**	**0.1%**	**0.1%**	**0.0%**	**0.0%**	0.0%	**2.0%**	**0.8%**	**0.8%**
Year*Water	0.0%	0.0%	0.0%	**0.1%**	**0.2%**	**0.1%**	0.0%	0.0%	0.0%	0.0%	**0.1%**	**0.2%**
Year*Variety	**0.7%**	**1.2%**	**1.5%**	**9.6%**	**0.7%**	**0.6%**	**0.6%**	**0.2%**	**0.1%**	**3.2%**	**1.1%**	**1.7%**
Sowing*Water	**0.2%**	**0.1%**	**0.1%**	0.0%	**0.3%**	**0.3%**	**0.3%**	**0.2%**	**0.1%**	**0.0%**	0.0%	0.0%
Sowing*Variety	**1.3%**	**1.3%**	**0.7%**	**2.0%**	**0.9%**	**1.2%**	**0.3%**	**0.3%**	**0.3%**	**0.7%**	**0.7%**	**1.3%**
Water*Variety	0.1%	0.1%	0.0%	**0.1%**	**0.1%**	**0.1%**	0.1%	0.0%	0.1%	**0.2%**	0.2%	0.3%

Year*Sowing*Water	**0.1%**	**0.1%**	**0.0%**	0.0%	**0.2%**	**0.1%**	**0.2%**	**0.0%**	0.0%	0.0%	0.0%	0.0%
Year*Sowing*Variety	**0.4%**	**0.3%**	**0.2%**	**0.9%**	**0.2%**	**0.2%**	**0.6%**	**0.3%**	**0.1%**	**0.4%**	**0.3%**	0.3%
Year*Water*Variety	**0.1%**	**0.1%**	0.0%	0.1%	**0.2%**	**0.2%**	0.1%	**0.1%**	**0.1%**	**0.2%**	**0.5%**	**0.9%**
Sowing*Water*Variety	0.1%	0.0%	0.0%	**0.2%**	0.0%	0.0%	0.1%	**0.1%**	**0.1%**	**0.2%**	**0.6%**	**0.9%**
Year*Sowing*Water*Variety	**0.1%**	**0.1%**	**0.1%**	0.1%	**0.1%**	**0.1%**	**0.2%**	**0.2%**	**0.2%**	0.1%	0.3%	0.5%

Tmin: minimum temperature (°C), TA: average temperature (°C), TM: maximum temperature (°C), PP: precipitation (mm), SR: global solar radiation (MJ m^{-2}), ETo: potential evapotranspiration (mm). * indicates that value of P V and GF were not normal and no transformation allowed to be converted into a normal distribution.

Table S4: Tukey's mean separation tests among genotypes for GY using the model with all 4 factors year, sowing, water and variety and all their interactions, by sowing date with Year, Water and Variety factors more interactions by water regimes with year, sowing and variety factors more interactions and by trial with only variety as factor. Variety ID number, least squared mean values and Letters (with correspondent groups), R^2 and p-values are shown.

Model with all 4 factors Year, Water, Sowing, Variety + interactions

Response GY

	R2 = 0.95	p-value<0.0001
Variety	Least Sq Mean	Letters
6	8.4	A
4	8.3	A
10	8.0	AB
7	7.9	ABC
9	7.4	ABCD
1	7.4	BCD
3	7.0	CD
2	6.8	D
5	5.3	E
8	5.3	E

1a SD	R2 = 0.95	p-value<0.0001

Model with 3 factors Year, Water, Variety + interactions

Variety	Least Sq Mean	Letters
4	9.6	A

6	9.3	AB
7	9.2	AB
10	8.5	AB
1	8.5	AB
9	8.4	AB
2	8.3	AB
3	7.9	BC
8	6.8	CD
5	6.4	D

2a SD R^2 = 0.92   p-value<0.0001

Model with 3 factors Year, Water, Variety + interactions

Variety	Least Sq Mean	Letters
10	7.5	A
6	7.5	A
4	7.0	AB
7	6.5	ABC
9	6.4	ABC
1	6.2	ABC
3	6.1	BC
2	5.3	CD
5	4.3	DE
8	3.9	E

Irrigated R^2 = 0.95   p-value<0.0001

Model with 3 factors Year, Sowing, Variety + interactions

Variety	Least Sq Mean	Letters
6	10.2	A
4	10.1	A
10	9.7	AB
7	9.5	AB
1	8.6	BC
3	8.6	BC
9	8.6	BC
2	8.0	CD
8	6.9	DE
5	6.4	E

Rainfed R2 = 0.93 p-value<0.0001

Model with 3 factors Year, Sowing, Variety + interactions

Variety	Least Sq Mean	Letters
4	6.6	A
6	6.5	A
10	6.3	A
9	6.3	A
7	6.2	A
1	6.2	A
2	5.5	AB
3	5.4	AB
5	4.2	BC
8	3.8	C

Response GY Trial=2021 LLEIDA 1 SD IRRIGATED

	R2 = 0.83	p-value<0.0001
Variety	Least Sq Mean	Letters
6	15.0	A
4	14.6	A
7	14.6	A
10	13.6	A
3	12.7	AB
1	12.5	AB
2	12.1	AB
9	11.9	ABC
8	9.7	BC
5	9.0	C

Response GY Trial=2021 LLEIDA 1 SD RAINFED

	R2 = 0.52	p-value<0.0503
Variety	Least Sq Mean	Letters
4	12.5	A
7	11.7	A
2	11.3	A
9	11.2	A
6	11.1	A
1	10.6	A
10	10.4	A

3	9.3	A
8	8.3	A
5	7.8	A

Response GY Trial=2021 LLEIDA 2 SD IRRIGATED

	R2 = 0.78	p-value<0.0001
Variety	Least Sq Mean	Letters
10	12.0	A
6	11.2	AB
4	11.1	AB
7	10.5	AB
3	9.4	ABC
9	8.9	ABC
1	8.5	ABC
2	7.6	BC
8	6.1	C
5	5.8	C

Response GY Trial=2021 LLEIDA 2 SD RAINFED

	R2 = 0.69	p-value<0.0015
Variety	Least Sq Mean	Letters
9	8.5	A
10	8.4	A
6	8.0	A
7	6.7	AB

1	6.6	AB
4	6.5	AB
2	6.1	AB
3	5.9	AB
5	5.1	AB
8	3.0	B

Response GY Trial=2022 LLEIDA 1 SD IRRIGATED

R2 = 0.46 p-value<0.1112

Variety	Least Sq Mean	Letters
4	7.4	A
9	7.4	A
6	7.3	A
2	7.0	A
1	7.0	A
7	6.9	A
10	6.7	A
8	6.4	A
5	6.2	A
3	6.1	A

Response GY Trial=2022 LLEIDA 1 SD RAINFED

R2 = 0.79 p-value<0.0001

Variety	Least Sq Mean	Letters
1	4.1	A

4	4.1	A
6	3.7	A
7	3.7	A
3	3.5	AB
10	3.4	AB
9	3.3	AB
8	2.6	B
2	2.6	B
5	2.5	B

Response GY Trial=2022 LLEIDA 2 SD IRRIGATED

	R2 = 0.75	p-value<0.0002
Variety	Least Sq Mean	Letters
6	7.3	A
4	7.2	A
1	6.5	AB
10	6.4	AB
3	6.1	ABC
9	6.1	ABC
7	6.0	ABC
2	5.4	BC
8	5.2	BC
5	4.8	C

Response GY Trial=2022 LLEIDA 2 SD RAINFED

	R2 = 0.86	p-value<0.0001
Variety	Least Sq Mean	Letters
1	3.4	A
4	3.4	A
6	3.3	AB
10	3.0	ABC
3	3.0	ABC
7	2.8	ABC
9	2.2	BCD
2	2.1	CD
5	1.3	D
8	1.1	D

Table S5: Pearson coefficients of genetic correlations (means of varieties) of hectoliter weight (HLW) with grain yield (GY), maximum temperature (TM), during the vegetative (V) and grain filling stages (GF), and days to heading (DH) are shown. Number of datapoints (Points) indicating the number of means of each genotype used in the analysis and the average (AVG) of all genotypes per trial are also shown. Significant values are indicated in bold font ($p<0.05$) and in normal text when non-significant ($p>0.05$).

Trials	HLW								AVG HLW	Points
	GY	AVG	TM V	AVG	TM GF	AVG	DH	AVG		
Trial 1	0.10	9.8	**-0.67**	13.4	NA	NA	**-0.68**	154.7	78.8	20
Trial 2	**0.67**	8.0	**-0.79**	14.4	NA	NA	**-0.80**	172.4	75.8	20
Trial 3	-0.17	11.2	**-0.39**	12.7	NA	NA	**-0.40**	182.4	78.8	36
Trial 4	0.19	5.5	**-0.35**	12.9	NA	NA	**-0.36**	189.4	74.0	36
Trial 5	-0.24	13.0	**-0.47**	14.9	NA	NA	**-0.49**	143.5	83.4	18
Trial 6	0.19	4.7	-0.16	14.6	NA	NA	-0.16	151.5	75.4	32
Trial 7	0.14	7.5	-0.11	13.5	NA	NA	-0.12	166.6	72.6	36
Trial 8	0.16	6.2	-0.08	14.1	NA	NA	-0.09	170.4	75.1	38
Trial 9	0.32	11.3	-0.09	13.8	NA	NA	-0.10	132.1	77.7	16
Trial 10	**0.75**	10.0	0.03	13.9	NA	NA	0.05	132.8	74.9	16

Trial 11	0.31	11.9	**-0.67**	13.8	**-0.68**	24.5	**-0.67**	145.1	77.8	22
Trial 12	-0.35	10.4	**-0.54**	13.8	**-0.56**	24.0	**-0.55**	145.1	80.6	22
Trial 13	0.20	7.3	0.16	15.7	0.25	21.7	0.12	138.9	78.7	18
Trial 14	0.16	7.6	-0.36	13.6	-0.33	21.7	-0.35	138.9	79.4	18
Trial 15	0.25	4.7	-0.15	13.4	-0.17	24.3	-0.18	162.0	78.4	36
Trial 16	**-0.38**	9.1	**-0.37**	13.5	NA	NA	**-0.38**	170.8	82.8	34
Trial 17	-0.41	8.8	**-0.55**	14.3	**-0.53**	30.1	**-0.53**	145.7	77.2	22
Trial 18	0.12	4.5	**-0.71**	14.3	**-0.66**	29.6	**-0.68**	146.8	72.0	22
Trial 19	-0.07	3.7	0.01	13.5	-0.05	30.0	-0.05	170.7	73.8	32
Trial 20	-0.42	6.3	**-0.49**	13.9	**-0.56**	28.2	**-0.48**	137.0	77.9	21
Trial 21	**0.34**	6.0	**-0.66**	18.3	**-0.43**	27.4	**-0.66**	108.6	74.4	159
Trial 22	**0.33**	4.4	**-0.56**	18.6	-0.09	27.3	**-0.54**	111.4	72.2	148
Trial 23	**0.50**	6.2	**-0.71**	18.2	**-0.39**	27.4	**-0.71**	107.3	73.7	99
Trial 24	**0.36**	7.3	**-0.67**	18.4	**-0.59**	27.5	**-0.67**	108.9	74.0	158
Trial 25	0.15	4.4	**-0.38**	18.6	0.03	27.2	**-0.35**	111.7	74.2	147

Trial 26	0.03	3.9	-0.29	15.4	0.09	24.7	-0.30	159.7	72.6	25

HLW: hectoliter weight (kg/hL), GY: grain yield (t ha^{-1}), TM V (during the vegetative stage) and GF (during the grain filling stage): maximum temperature (°C), DH: days to heading (days).

Table S6. Pearson correlation coefficients of growing degree days to heading, along with average values (AVG), with plant traits and grain quality parameters, per trial and averaging all trials, are shown. Number of datapoints (Points) indicating the number of means of each genotype used in the analysis per trial are also shown. Significant correlations are indicated in bold font (p<0.05) and normal format indicates non-significant correlations at p>0.05.

Trials	GDD (DH)									AVG GDD (DH)	Points
	**GY**	**Prot (%)**	**HLW**	**TKW**	**NG**	**W**	**P/L**	**P**	**L**		
2021 Almacelles 1SD Irrigated	**-0.85**	**0.72**	**-0.91**	**-0.77**	-0.20	-0.31	**-0.82**	**-0.65**	**0.77**	1261.5	10
2021 Almacelles 2SD Irrigated	**-0.92**	**0.89**	**-0.83**	**-0.64**	**-0.69**	-0.08	-0.56	-0.44	0.57	1255.2	10
2021 Almacelles 1SD Rainfed	-0.61	0.43	**-0.88**	**-0.70**	0.01	-0.41	**-0.70**	**-0.73**	0.63	1246.1	10
2021 Almacelles 2SD Rainfed	-0.62	**0.74**	**-0.88**	**-0.66**	-0.32	-0.43	-0.60	-0.61	0.57	1193.1	10
2022 Sucs 1SD Irrigated	-0.29	0.20	**-0.73**	-0.33	0.14	-0.17	**-0.65**	-0.45	0.61	1240.7	10
2022 Sucs 2SD Irrigated	**-0.81**	0.59	**-0.86**	-0.59	-0.29	-0.45	**-0.78**	**-0.78**	**0.66**	1223.1	10
2022 Sucs 1SD Rainfed	**-0.94**	**0.96**	**-0.71**	-0.17	**-0.74**	-0.22	-0.57	-0.52	**0.76**	1224.4	10
2022 Sucs 2SD Rainfed	**-0.95**	**0.86**	-0.60	**0.65**	**-0.96**	-0.44	-0.46	-0.55	**0.64**	1194.1	10
*Irrigated 1SD	**-0.79**	0.57	**-0.85**	**-0.64**	-0.10	-0.30	**-0.78**	-0.61	**0.79**	1251.1	10
*Irrigated 2SD	**-0.90**	**0.82**	**-0.86**	-0.63	-0.59	-0.27	**-0.74**	**-0.64**	**0.68**	1239.1	10

*Rainfed 1SD	**-0.75**	**0.88**	**-0.82**	-0.53	-0.29	-0.38	**-0.68**	**-0.69**	**0.78**	1235.2	10
*Rainfed 2SD	**-0.75**	**0.84**	**-0.80**	-0.05	**-0.69**	-0.48	-0.61	**-0.68**	**0.70**	1193.6	10
Averages all trials	**-0.85**	**0.82**	**-0.84**	-0.53	-0.48	-0.39	**-0.74**	**-0.70**	**0.79**	1229.8	10

GDD (DH): growing degree days to heading (°Cd), GY: grain yield (t ha^{-1}), Prot (%): grain protein concentration (%), HLW: hectoliter weight (kg/hL), TKW: thousand kernel weight (g/1000 kernels), NG: number of grains m^{-2} (units m^{-2}), W: dough strength (10^{-4} J), P/L: tenacity/extensibility ratio, P: tenacity (mm), L: extensibility (mm), * indicates correlations performed by averaging the two years of trials.
